# Effects of Moloney Leukemia Virus 10 Protein on Hepatitis B Virus Infection and Viral Replication

**DOI:** 10.3390/v11070651

**Published:** 2019-07-17

**Authors:** Maritza N. Puray-Chavez, Mahmoud H. Farghali, Vincent Yapo, Andrew D. Huber, Dandan Liu, Tanyaradzwa P. Ndongwe, Mary C. Casey, Thomas G. Laughlin, Mark Hannink, Philip R. Tedbury, Stefan G. Sarafianos

**Affiliations:** 1 Christopher S. Bond Life Sciences Center, University of Missouri, Columbia, MO 65211, USA; 2 Department of Molecular Microbiology and Immunology, School of Medicine, University of Missouri, Columbia, MO 65212, USA; 3 Department of Pharmaceutical Microbiology, Faculty of Pharmacy, Tanta University, Tanta 31512, Egypt; 4 Department of Veterinary Pathobiology, College of Veterinary Medicine, University of Missouri, Columbia, MO 65211, USA; 5 Department of Biochemistry, University of Missouri, Columbia, MO 65211, USA

**Keywords:** hepatitis B virus, moloney leukemia virus 10, host factors, gene expression

## Abstract

Moloney leukemia virus 10 (MOV10) is an RNA helicase that has been shown to affect the replication of several viruses. The effect of MOV10 on Hepatitis B virus (HBV) infection is not known and its role on the replication of this virus is poorly understood. We investigated the effect of MOV10 down-regulation and MOV10 over-expression on HBV in a variety of cell lines, as well as in an infection system using a replication competent virus. We report that MOV10 down-regulation, using siRNA, shRNA, and CRISPR/Cas9 gene editing technology, resulted in increased levels of HBV DNA, HBV pre-genomic RNA, and HBV core protein. In contrast, MOV10 over-expression reduced HBV DNA, HBV pre-genomic RNA, and HBV core protein. These effects were consistent in all tested cell lines, providing strong evidence for the involvement of MOV10 in the HBV life cycle. We demonstrated that MOV10 does not interact with HBV-core. However, MOV10 binds HBV pgRNA and this interaction does not affect HBV pgRNA decay rate. We conclude that the restriction of HBV by MOV10 is mediated through effects at the level of viral RNA.

## 1. Introduction

Chronic Hepatitis B virus (HBV) infection is one of the most serious public health problems worldwide. According to the World Health Organization, in 2015, the number of chronic HBV patients was approximately 257 million people. Moreover, the number of deaths from complications of chronic HBV infection, such as hepatocellular carcinoma, reached 887,000. HBV is an enveloped DNA virus. Its genome is composed of partially double-stranded, relaxed-circular DNA (rcDNA), which is converted in the nucleus of infected cells to a plasmid-like, covalently closed circular DNA (cccDNA). HBV cccDNA serves as a template for HBV RNA synthesis [1,2]. One of those RNA products is the pre-genomic RNA (pgRNA), which encodes the HBV polymerase and HBV core proteins. HBV polymerase and pgRNA are encapsidated by the core protein, where the viral polymerase synthesizes the minus-strand DNA. In addition to its DNA synthesis function, the viral polymerase also has an RNase H function that degrades the pgRNA template during DNA synthesis. The minus-strand DNA product of first strand synthesis is then used by the viral polymerase as a template to synthesize the partially completed plus-strand DNA [1,2,3].

The current available treatments for chronic HBV infection include immunomodulators, such as interferon alpha, and nucleos(t)ide analogues, such as tenofovir and entecavir [4,5]. However, the viral core protein and the RNase H function of HBV polymerase may also be targets for drug development [6,7,8,9,10,11,12]. Interferon alpha treatment can cure chronic HBV infection, but only in a minority of cases (30–40%) [13]. It is also expensive and has several side effects that limit its use [5,13]. Nucleos(t)ide analogues have fewer side effects, but life-long treatment is usually required to maintain the suppression of viral load [5]. Therefore, new strategies for chronic HBV treatment are required.

Studying host factors that have a role in HBV infection and replication may contribute to new strategies to cure chronic HBV infection. Host factors such as Apolipoprotein-B-mRNA-editing enzyme catalytic polypeptide-like 3 (APOBEC3), DEAD-box helicase 3 X-linked (DDX3), Myxovirus resistance protein 1 (MxA), and a wide range of cytokines have been shown to affect HBV replication [14,15,16,17]. A recent study suggested that Moloney leukemia virus 10 (MOV10) may affect HBV replication in the hepatoma cell lines HepG2 and HepG2.2.15 [18]. We sought to build on the findings of that study by examining the effects of MOV10 in a more physiologically relevant system, using an infectious virus. MOV10 is a DExD-box RNA helicase and a reported component of cytoplasmic RNA processing centers known as P-bodies [19]. Its helicase activity unwinds RNA in an ATP-dependent manner and in a 5′ to 3′ direction [20]. MOV10 is important for the function of the RNA-induced silencing complex (RISC) [21]. Previous studies reported that MOV10 can restrict a wide range of viruses, including retroviruses. Wang et al. reported that over-expression of MOV10 suppresses infection by human immunodeficiency virus type 1 (HIV-1), murine leukemia virus (MLV), and simian immunodeficiency virus (SIV), while MOV10 down-regulation results in enhanced HIV-1 infectivity and replication [22]. Burdick et al. demonstrated that MOV10 over-expression reduces HIV-1 production and infectivity, while MOV10 down-regulation decreases HIV-1 production but not infectivity [23]. Furtak et al. showed that MOV10 knockdown results in the production of HIV-1 particles with less infectivity, and that MOV10 over-expression reduces HIV-1 infectivity [24]. Previous studies showed that MOV10 can be incorporated in HIV-1 virions [22,23]. It was also reported that MOV10 and HIV-1 RNA co-immunoprecipitated, and that MOV10 over-expression reduces the products of late reverse transcription of HIV-1 [23]. The effect of MOV10 on HIV reverse transcription raises the possibility that it may also have a role in the life cycle of HBV, which is another virus that also uses a reverse transcriptase in its life cycle to reverse transcribe an RNA intermediate, pgRNA, into DNA. The specific mechanism by which MOV10 affects viral replication is not fully understood. Apolipoprotein B mRNA editing enzyme, catalytic polypeptide-like (APOBEC) 3G or (A3G) also localizes to P-bodies, and interacts with MOV10 [25]. In addition, it was reported that MOV10 inhibits the proteasomal degradation of A3G, a cytidine deaminase, which can restrict HIV-1 infection [26]. Cuevas et al. reported that MOV10 has an antiviral activity against RNA viruses, mediated by enhancing interferon (IFN) production, independently from the Retinoic acid-inducible gene I/ mitochondrial antiviral-signaling protein (RIG-I/MAVS) RNA-sensing pathway [27]. Schoggins et al. demonstrated that MOV10 has a restriction activity against Hepatitis C virus (HCV) [28]. Furthermore, MOV10 down-regulation resulted in the inhibition of Hepatitis D virus (HDV) replication [29]. Previous studies reported that DDX3, similar to MOV10, also colocalizes with P-bodies and RISC [30]. DDX3 was shown to restrict the transcription of HBV from cccDNA [16,30]. Recently, Ma et al. tested the potential effect of MOV10 on HBV replication using the HepG2 and HepG2.2.15 cell lines [18]. They reported a complex effect in their experimental system: the transfection of low doses of ectopic MOV10 appeared to enhance HBV expression, while MOV10 down-regulation and transfection of high doses of ectopic MOV10 decreased HBV expression. They concluded that there is a putative, yet complicated role of MOV10 in HBV replication. However, the role of MOV10 in other cell lines, and more importantly, in the more biologically relevant system of authentic infection by fully infectious viruses, was not addressed.

To address the role of MOV10 in HBV infection, we generated MOV10 over-expression and MOV10 knockdown stable cell lines to investigate the role of MOV10 in HBV replication. We tested the effect of MOV10 over-expression and down-regulation in different systems, including the HepAD38 stable cell line that can be induced to express HBV [31], transfection of HepG2 cells, and infection of cells that express the HBV receptor, HepG2-sodium taurocholate cotransporting polypeptide (NTCP) [32], by monitoring a broad set of HBV markers that included intracellular HBV DNA, HBV RNA, and HBV core protein. We found that MOV10 over-expression restricted HBV replication, while MOV10 down-regulation enhanced HBV replication. These results were consistent across all of the systems we used. We also demonstrated binding of MOV10 to HBV pgRNA, although we were not able to detect an effect of MOV10 expression on the HBV pgRNA decay rate. Our results provide comprehensive evidence for an inhibitory role of MOV10 during HBV replication. This inhibition appears to be mediated via an activity of MOV10 targeting HBV pre-genomic RNA that remains to be clearly elucidated.

## 2. Materials and Methods

### 2.1. Cell Lines

HepAD38 is an inducible hepatoblastoma cell line that contains an HBV infectious clone under the control of a tetracycline (tet)-off regulated cytomegalovirus (CMV) promoter; HBV is induced when tet is removed from the culture medium [31]. HepG2 cells were purchased from the American Type Culture Collection (Manassas, VA, USA). HepG2-NTCP clone 3E8 and HepG2-NTCP clone 7 cells were gifts from Drs. Charles Rice and Eleftherios Michailidis (Rockefeller University, NY, USA). HepG2-NTCP clone 3E8 and HepG2-NTCP clone 7 cells were described previously [33,34]. Human 293T cells were from the American Type Culture Collection. In general, all cells were maintained in Dulbecco’s Modified Eagle’s Medium (DMEM) with 10% fetal bovine serum (FBS). For HepAD38 cells, 0.3 µg/mL tet and 400 µg/mL G418 were added to the culture medium, as previously described [31]. Cells were incubated at 37 °C in the presence of 5% CO_2_.

### 2.2. Plasmids

The HBV genome harboring plasmid pCMV-HBV-LE-II (pHBV) was a gift from John E. Tavis (Saint Louis University, MO, USA). Core protein (Cp)-deficient pHBV was constructed by introducing a premature stop codon to Cp in pHBV by site-directed mutagenesis with primers 5′-GGAGATCTCAGACGGAAGGAAAGAAGTC-3′ and 5′-GACTTCTTTCCTTCCGTCTGAGATCTCC-3′. The HBV core expressing plasmid (pC183) was described previously [35]. The eYFP-MOV10 plasmid was described previously [23]. The eYFP-empty plasmid was produced from eYFP-MOV10 by cutting out the MOV10 sequence.

### 2.3. Virus Production and Infection

HBV was produced as previously described [33,36], with modifications. Briefly, HepAD38 cells were maintained in DMEM supplemented with 10% FBS, 0.3 µg/mL tet, and 400 µg/mL G418 until cells reached 60–70% confluency, then the culture medium was replaced with DMEM/3% FBS without antibiotics. The removal of tet activated the transcription of HBV pgRNA. Supernatant was collected every 3–4 days, then the supernatant was clarified by centrifugation at 3000× *g* at 4 °C for 10 min, and filtration (0.22 µm polyethersulphone). Filtered supernatants were stored at −80 °C. HBV was concentrated (~100-fold) using Amicon Ultra-15 Centrifugal Filter Unit (Catalogue number: UFC910024), resuspended in cold DMEM, aliquoted, and stored at −80 °C.

HBV infection was performed as previously described [33,37], with a modified DMSO concentration for HepG2-NTCP clone 3E8 infection. HepG2-NTCP clone 3E8 cells were treated with 1.5% DMSO (Sigma-Aldrich) for at least 24 h before infection. Cells were inoculated with HBV in the presence of 1.5% DMSO, 3% FBS, 4% PEG 8000, and 0.1 mM non-essential amino acids (NEAA) (Gibco, Gaithersburg, MD, USA), and spinoculated at 1000 × *g* for 1 h at room tempeature (RT), then incubated at 37 °C. After 24 h, cells were washed four times with DPBS, then cells were washed once and kept in a fresh medium of DMEM containing 4% PEG 8000, 1.5% DMSO, 3% FBS, and 0.1 mM NEAA. The same steps were followed for infecting HepG2-NTCP clone 7, except that the DMSO concentration used was 2%.

### 2.4. Generation of Stable Cell Lines with MOV10 Knocked Out, Down-Regulated, or Over-Expressed

The stable cell lines were generated as previously described [38]. HepG2-NTCP cells were transduced with pseudotyped lentiviral particles, expressing MOV10-shRNA or HA-MOV10 constructs. Puromycin was used to select the transduced cells. To generate HepG2 cells in which MOV10 was knocked out, CRISPR/Cas9 gene editing technology was used. The plasmid harboring Cas9, guide-RNA (gRNA), and GFP (pCas9-GFP-Mov10_T1) was purchased from OriGene Technologies. The gRNA targets Cas9 to MOV10 gene 5′ end (5′-TGTCCAGTCCCCGAACGACC-3′). HepG2 cells were transfected with pCas9-GFP-Mov10_T1 plasmid using Fugene 6 reagent (Promega, Madison, WI, USA). The ratio of Fugene (µL): plasmid (µg) was 3:1. Fluorescence activated cell sorting was used to sort GFP-positive cells, which were then allowed to expand to form individual clones. Expression of the CRISPR/Cas9 machinery was transient in this system. Western blotting was used to detect the level of MOV10 expression in the resulting individual clones, and the MOV10 loci were sequenced to confirm the presence of a frame shift mutation in both loci.

### 2.5. Transfection

HepG2 cells (2 × 10^5^) were seeded in 12-well plates coated with collagen. The cells were transfected with pHBV plasmid (0.4 µg/well) using Fugene 6 reagent (Promega, Madison, WI, USA). The ratio of Fugene (µL): plasmid (µg) was 3:1. MOV10 siRNA (catalogue number: L-014162-00) and non-targeting control siRNA (catalogue number: D-001810-10) were purchased from Dharmacon as pools of four siRNAs targeting distinct sites (SMARTpool siRNA). Non-targetting siRNA controls were used as they would induce the RNAi machinery, unlike an untransfected control. Cells were transfected using Lipofectamine RNAiMAX (Invitrogen, Carlsbad, CA, USA) following the manufacturer’s instructions. Human 293T cells were transfected with the indicated plasmids using Fugene 6 reagent (Promega, Madison, WI, USA). The ratio of Fugene (µL): plasmid (µg) was 3:1.

### 2.6. qPCR Analysis of HBV DNA

The QIAamp DNA Blood Mini Kit (Qiagen) was used to extract total cellular DNA. Sequences of forward and reverse primers used for HBV DNA quantification were 5’-CCTGGTTATCGCTGGATGTGT-3’ (nt 364 to 384) and 5’-GGACAAACGGGCAACATACCTT-3’ (nt 479 to 458), respectively. These primers targeted conserved regions in the HBV S gene [35,39]. HBV DNA was quantified using ABsolute qPCR SYBR green mix (Thermo Scientific, Waltham, MA, USA) or Applied Biosystems™ PowerUp™ SYBR™ Green Master Mix, in a PikoReal real-time PCR system. Equal amounts of DNA were loaded per sample. A reaction volume of 10 µL was heated to 50 °C for 2 min, followed by denaturation at 95 °C for 2 min, followed by 40 cycles of denaturation at 95 °C for 15 s, annealing at 60 °C for 15 s, and extension at 72 °C for 1 min, according to manufacturer’s instructions. Cycle threshold (CT) values for HBV amplification were normalized to the cellular gene beta-globin CT. Beta-globin primers were LA1 5’-ACACAACTGTGTTCACTAGC-3’ and LA2 5’-CAACTTCATCCACGTTCACC-3’ [40]. A standard curve generated with serial dilutions of pHBV was used to determine HBV DNA copy numbers.

### 2.7. Quantification of HBV pgRNA

Cells were pelleted and lysed in 300 µL of TRI Reagent (Sigma-Aldrich, St. Louis, MO, USA). Total cellular RNA was extracted according to the manufacturer’s instructions. Molecular biology grade glycogen (Thermo Scientific, Waltham, MA, USA) was added to isopropanol in the RNA precipitation step to aid visualization of the RNA pellet. HBV pgRNA was quantified using the Power SYBR^®^ Green RNA-to-CT™ 1-Step Kit (Applied Biosystems, Foster City, CA, USA) in a PikoReal real-time PCR system. Forward and reverse primers used for pgRNA amplification were 5′-GAGTGTGGATTCGCACTCC-3′ (nt 2270–2288) and 5′-GAGGCGAGGGAGTTCTTCT-3′ (nt 2392–2374), respectively [32]. These primers would recognize both the pgRNA and the pre-Core RNA. However, pgRNA was by far the more abundant species [41,42]. The bias in favor of pgRNA was likely even more exaggerated in our system, as the timing of the experiments would not allow for extensive cccDNA formation. Cycle threshold (CT) values were normalized to glyceraldehyde 3-phosphate dehydrogenase (GAPDH) CT values. The GAPDH forward and reverse primers were 5′-CGCTCTCTGCTCCTCCTGTTC-3′ and 5′-CGCCCAATACGACCAAATCCG-3′, respectively. To test the effect of MOV10 over-expression on HBV pgRNA degradation rate, the human 293T cells’ total RNA was extracted. Reverse transcription of HBV pgRNA and RPL18A mRNA (normalization control) was performed using High-Capacity cDNA Reverse Transcription Kit (Applied Biosystems, Foster City, CA, USA) according to the manufacturer’s instructions. HBV pgRNA and RPL18A reverse primers were used for the synthesis of cDNAs. cDNAs were then amplified and quantified using Applied Biosystems™ PowerUp™ SYBR™ Green Master Mix, in Bio-Rad CFX Connect Real-Time PCR Detection System. CT values for HBV pgRNA cDNA amplification were normalized to RPL18A. The forward and reverse primers of RPL18A were 5′- GGAGAGCACGCCATGAAG-3′ and 5′- AAGATTCGCATGCGGTAGAG-3′, respectively [20].

### 2.8. Western Blotting

Cells were lysed using a radioimmunoprecipitation assay buffer (50 mM Tris/HCl, 150 mM NaCl, 1 mM EGTA, 1 mM EDTA, 0.1% sodium dodecyl sulfate (SDS), 1% Triton X-100, pH 7.5) to which protease inhibitor cocktail (Sigma-Aldrich) was added. The protein concentration was measured using Bradford assay (Bio-Rad). A total of 10–20 µg protein was separated by SDS-polyacrylamide gel electrophoresis (SDS-PAGE) and transferred to polyvinylidene difluoride Immobilon-P membranes (MilliporeSigma, Burlington, MA, USA). Membranes were blocked with 5% non-fat milk in Tris Buffered Saline (TBS) with 0.1% Tween 20 (TBST). Membranes were probed with a rabbit anti-HBV core antibody (Austral Biologicals HBP-023-9, San Ramon, CA, USA) 1:500 dilution, rabbit anti-MOV10 antibody (Abcam, ab80613, Cambridge, MA, USA) 1:2,000 dilution, overnight at 4 °C, or mouse anti-GAPDH antibody (Santa Cruz Biotechnology sc-365062) 1:10,000 dilution at RT for 1 h, then anti-mouse (Sigma-Aldrich A9044, St. Louis, MO, USA) and anti-rabbit (Sigma-Aldrich A0545, St. Louis, MO, USA) horseradish peroxidase (HRP)-conjugated secondary antibodies were added at RT for 1 h. A Luminata Forte Western HRP substrate (MilliporeSigma, Burlington, MA, USA) was added to the membrane. Then, images were taken using a UVP BioSpectrum 815 imaging system (Analytic Jena, Upland, CA, USA).

### 2.9. RNA-Immunoprecipitation

The RNA-immunoprecipitation experiment protocol was adapted from Keene et al. [43]. Briefly, HepG2-NTCP clone 7 cells were infected with HBV for five days. At the time of harvest, formaldehyde was added to the culture medium for 10 min to achieve crosslinking at a final concentration of 1%. The crosslinking reaction was quenched with glycine for 10 min at a final concentration of 125 mM. Fixed cells were washed twice with ice-cold PBS, scraped out of the culture dish with a cell scraper, and recovered in sterile PBS. At least 6.5 million cells were used per immunoprecipitation (IP) reaction.

The cells were pelleted at 1000× *g* for 10 min at 4 °C, and lysed on ice for 5 min with polysome lysis buffer (100 mM KCl, 5 mM MgCl_2_, 10 mM HEPES pH 7.0, 0.5% NP40, 1 mM DTT, 400 µM Vanadyl Ribonucleoside Complexes, nuclease-free water) supplemented with RNase and protease inhibitor cocktails. Lysis was completed with a freeze–thaw step, wherein samples were incubated in liquid nitrogen overnight. Protein G beads (Invitrogen, Carlsbad, CA, USA) were coated overnight with 6 µg per IP of mouse anti-MOV10 (ab176687, Abcam, Cambridge, MA, USA) or mouse normal IgG conjugated to agarose (sc-2343, Santa Cruz, Dallas, TX, USA). The next day, lysates were thawed on ice for about an hour, and cleared at 15,000× *g* for 15 min at 4 °C. Cleared lysates were split into an input sample (10% of total clear lysate), an MOV10 IP sample (first half of remaining lysate), and an IgG IP sample (second half). Input samples were frozen at –80 °C, whereas antibody-coated beads were added to corresponding IP samples. IP samples + antibody-coated beads samples were incubated for 2 h at RT, and washed five times with an ice-cold mixture of 1% urea + NT2 buffer (50 mM Tris-HCl pH 7.4, 150 mM NaCl, 1 mM MgCl_2_, 0.05% IGEPAL CA-630 [Nonidet P-40 substitute], nuclease-free water). Ribonucleoproteins complexes in the IP and input samples were treated with 100 µL of NT2 buffer, supplemented with proteinase K for 30 min at 55 °C.

RNA cleanup, including in-column DNase digestion, was performed with the RNeasy mini kit (Qiagen). Ten microliters of each RNA sample underwent cDNA synthesis in a total volume of 20 µL using the High Capacity cDNA Reverse Transcription Kit (Invitrogen) and random hexamer primers, as follows: 25 °C for 10 min (step 1), 37 °C for 2 h (step 2), 85 °C for 5 min (step 3), and 4 °C ∞ (step 4). cDNA samples were quantified with the PowerUp SYBR Green qPCR kit (Thermo Fischer Scientific, Waltham, MA, USA) against a standard curve using a set of primers against HBV pgRNA or against human GAPDH mRNA. The qPCR conditions were as follows: 50 °C for 5 min (step 1), 95 °C for 2 min (step 2), 35 cycles of 95 °C for 15 s, 60 °C for 30 s, and 72 °C for 1 min (step 3).

### 2.10. Co-Immunoprecipitation

Co-immunoprecipitation was performed as previously described [26,44], with modifications. Briefly, human 293T cells were lysed for 30 min at 4 °C, using the lysis buffer (150 mM NaCl, 50 mM Tris–HCl, pH 7, 1 mM EDTA, 0.5% IGEPAL, 1% Triton X-100), to which protease inhibitor cocktail (Sigma-Aldrich, St. Louis, MO, USA) was added; subsequently, the cell lysates were clarified. Immunoprecipitation was performed using Dynabeads™ Protein G (Catalogue number: 10004D) according to the manufacturer’s instructions. Mouse anti-HBV core (Thermo Fischer Scientific MA1-7606, Waltham, MA, USA) or mouse anti-MOV10 (Abcam ab 176687, Cambridge, MA, USA) antibodies were incubated with Dynabeads™ Protein for 24 h at 4 °C with rotation. Cell lysates were then mixed with the beads and incubated for 18–24 h at 4 °C with rotation. Where indicated, samples were incubated with RNase A (Qiagen, Germantown, MD, USA) (5 mg/mL) for 60–90 min at 4 °C prior to immunoprecipitation. Immunoprecipitates were then analyzed by SDS-PAGE followed by western blotting. For western blotting, membranes were probed with rabbit anti-HBV core antibody (Austral Biologicals HBP-023-9), 1:500 dilution, and rabbit anti-MOV10 antibody (Abcam, ab80613, Cambridge, MA, USA), 1:1000 dilution, overnight at 4 °C.

### 2.11. Statistics

Statistical analyses were performed and graphs plotted using Graphpad Prism. One phase decay curves were plotted for RNA decay, fixing y 0 to 100 and the plateau to 0.

## 3. Results

### 3.1. Down-Regulation of MOV10 Enhances HBV Gene Expression in HepAD38 Cells

**Figure 1:**
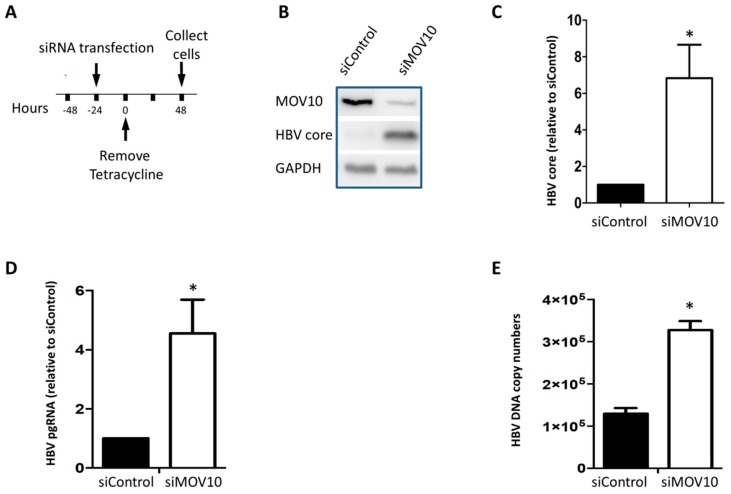
Down-regulation of MOV10 (Moloney Leukemia Virus 10) enhances Hepatitis B virus (HBV) gene expression in HepAD38 cells. (**A**) Time course of the experiment. HepAD38 cells were transfected with 50 nM siMOV10 or siControl 24 h before tetracycline (tet) removal. Cells were collected two days after tet removal. (**B**) Western blot showing HBV core and MOV10 expression in siRNA treated cells. Glyceraldehyde 3-phosphate dehydrogenase (GAPDH) was used as a loading control. (**C**) Quantification of HBV core expression determined by western blotting. (**D**) Intracellular HBV pgRNA level determined by RT-qPCR. Data were normalized to GAPDH mRNA expression. (**E**) Intracellular HBV DNA level determined by qPCR. Data represent mean ± standard deviation from two independent experiments. * *p*-value < 0.05, two-tailed unpaired *t*-test.

We first determined the influence of endogenous MOV10 on HBV gene expression. HepAD38 cells produce HBV under the control of a tet-regulated promoter (tet-off system) [31]. When tet is removed from the culture medium, HBV RNA transcription and protein synthesis are induced. We used a pool of four siRNAs to down-regulate MOV10 expression. Cells were transfected with 50 nM MOV10 siRNA (siMOV10) or the non-targeting control siRNA (siControl) one day before tet was removed from the culture medium. Two days after the removal of tet, the cells were collected (Figure 1A). We performed western blotting to determine levels of MOV10, HBV core protein, and GAPDH as a loading control (Figure 1B,C). HBV core protein level was significantly elevated in the siMOV10 treated cells compared to the cells treated with siControl. Total intracellular RNA was extracted and HBV pgRNA was measured by reverse transcription quantitative PCR (RT-qPCR). Our results show that HBV pgRNA level was significantly increased in the cells treated with siMOV10 compared to the cells treated with siControl (Figure 1D). Intracellular DNA was extracted and HBV DNA was quantified by quantitative PCR (qPCR). Like other HBV markers, MOV10 down-regulation resulted in a significant increase in intracellular HBV DNA (Figure 1E).

### 3.2. Down-Regulation of MOV10 Enhances HBV Gene Expression in HBV-Transfected HepG2 Cells

**Figure 2:**
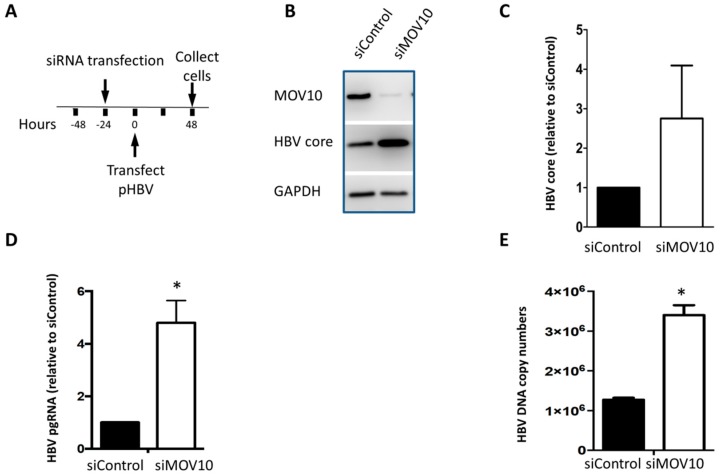
Down-regulation of MOV10 enhances HBV gene expression in HepG2 cells. (**A**) Time course of the experiment. HepG2cells were transfected with 20 nM siMOV10 or siControl 24 h before transfection with pHBV plasmid. Cells were collected two days after HBV plasmid transfection. (**B**) Western blot showing HBV core and MOV10 expression in siRNA treated cells. GAPDH was used as a loading control. (**C**) Quantification of HBV core expression determined by western blotting. (**D**) Intracellular HBV pgRNA level determined by RT-qPCR. Data were normalized to GAPDH mRNA expression. (**E**) Intracellular HBV DNA level determined by qPCR. Data represent mean ± standard deviation from two independent experiments. * *p*-value < 0.05, two-tailed unpaired *t*-test.

**Figure 3:**
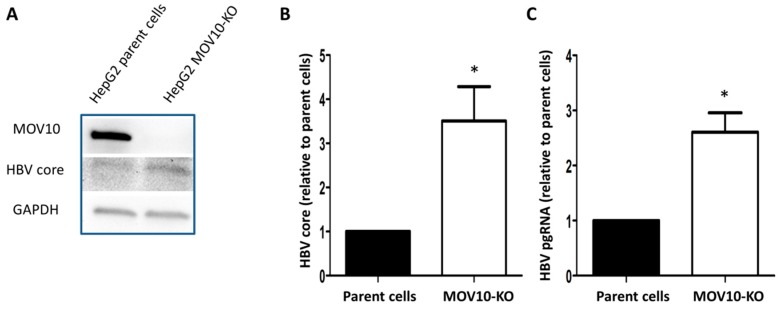
HBV gene expression in HepG2 MOV10-KO cells. (**A**) Western blot showing HBV core and MOV10 in HepG2 MOV10-KO cells compared to HepG2 parent cells. GAPDH was used as a loading control. (**B**) Quantification of HBV core expression determined by western blotting. (**C**) Intracellular HBV pgRNA level determined by qPCR after cDNA synthesis. Data were normalized to RPL18A mRNA expression. Data represent mean ± standard deviation from two independent experiments. * *p*-value < 0.05, two-tailed unpaired *t*-test.

To validate the observation that down-regulating MOV10 enhances HBV gene expression, we performed a similar experiment using a transient transfection of HepG2 cells with a plasmid clone of HBV. The cells were transfected with 20 nM of siMOV10 or siControl. The next day, the cells were transfected with pHBV. Cells were collected two days after transfection (Figure 2A). MOV10 down-regulation was confirmed with western blotting (Figure 2B). Consistent with our AD38 results, the HBV core protein level was increased in the cells treated with siMOV10 compared to the siControl-treated cells (Figure 2C) in the HepG2 transfection system. Again, we found that MOV10 down-regulation also resulted in significantly increased levels of HBV pgRNA and HBV DNA (Figure 2D,E). To confirm our findings, we used CRISPR/Cas9 technology to generate a stable HepG2 cell line, in which the MOV10 gene was knocked-out (HepG2 MOV10-KO). HepG2 parent cells and HepG2 MOV10-KO cells were transfected with pHBV. The cells were collected three days after the plasmid transfection. The suppression of MOV10 was confirmed with western blotting (Figure 3A). We found that HBV core protein levels were increased in HepG2 MOV10-KO cells relative to parent cells (~3.5-fold increase) (Figure 3B). HBV pgRNA was also significantly increased in HepG2 MOV10-KO cells (Figure 3C). Collectively, these findings demonstrate that MOV10 down-regulation enhances HBV gene expression and capsid production.

### 3.3. Down-Regulation of MOV10 Enhances HBV Infection of HepG2-NTCP-C7 Cells

**Figure 4:**
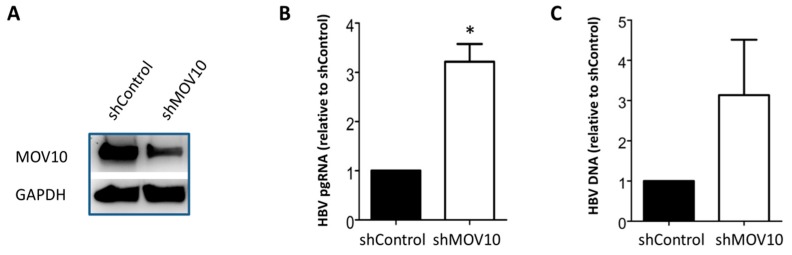
Down-regulation of MOV10 enhances HBV infection of HepG2-sodium taurocholate co-transporting polypeptide (NTCP) cells. HepG2-NTCP-C7 stable cell lines, shControl, and shMOV10, were infected with 250 HBV genome equivalents per cell. Five days later, cells were collected. (**A**) Western blotting showing MOV10 expression in shMOV10 stable cell line compared to shControl. GAPDH was used as a loading control. (**B**) Intracellular HBV pgRNA level determined by RT-qPCR. Data were normalized to GAPDH mRNA expression. (**C**) Intracellular HBV DNA level determined by qPCR. Data represent mean ± standard deviation from at least two independent experiments. * *p*-value < 0.05, two-tailed unpaired *t*-test.

Our results thus far have shown that MOV10 down-regulation enhanced HBV gene expression in transfection and stable cell systems. We next investigated the effect of MOV10 down-regulation on HBV in an infection system. For this purpose, we used HepG2-NTCP-C7 cells that expressed the HBV receptor NTCP [33]. Using shRNA-lentiviral vectors, we generated a stable MOV10 knock-down cell line (shMOV10) and a cell line transduced with a scrambled shRNA construct (shControl) as a control. Cells were infected with 250 HBV genome equivalents per cell. Five days later, the cells were collected, and proteins, intracellular RNA, and DNA were extracted. The level of MOV10 in knock-down cells was reduced by ~73%, as determined by western blot analysis (Figure 4A). HBV pgRNA and HBV DNA levels were measured using RT-qPCR and qPCR, respectively. Our results showed that, compared to control cells, HBV pgRNA levels were significantly increased in shMOV10 cells (~3-fold increase) (Figure 4B). HBV DNA production was also increased in shMOV10 cells (Figure 4C). These data indicate that MOV10 suppression enhanced HBV gene expression following infection.

### 3.4. Over-Expression of MOV10 Diminishes HBV Infection of HepG2-NTCP-3E8 Cells

**Figure 5:**
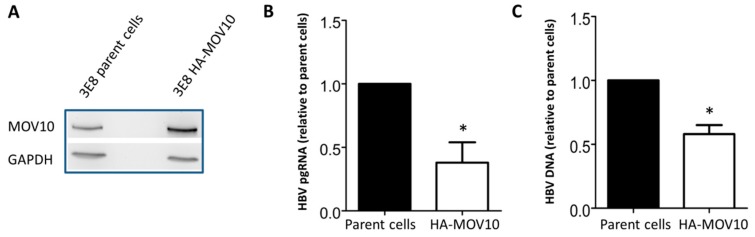
MOV10 over-expression diminishes HBV infection of HepG2-NTCP cells. HepG2-NTCP-3E8 parent cells and HepG2-NTCP-3E8 HA-MOV10 cells were infected with 400 HBV genome equivalents per cell. Five days later, cells were collected. (**A**) Western blot showing MOV10 expression in 3E8 HA-MOV10 stable cell line compared to HepG2-NTCP-3E8 parent cells. GAPDH was used as a loading control (**B**) Intracellular HBV pgRNA level determined by RT-qPCR. Data were normalized to GAPDH mRNA. (**C**) Intracellular HBV DNA level determined by qPCR. Data represent mean ± standard deviation from two independent experiments. * *p*-value < 0.05.

Having shown that MOV10 suppression enhanced HBV gene expression, we moved onto examining the effects of MOV10 over-expression. We used HepG2-NTCP clone 3E8 cells, which expressed the HBV receptor NTCP [34], to generate a stable cell line that over-expressed MOV10 (3E8 HA-MOV10). These cells were infected with 400 HBV genome equivalents per cell. Five days post-infection, cells were collected and the protein, RNA, and DNA harvested. MOV10 over-expression was confirmed by western blot (Figure 5A). We also attempted western blotting for HBV Cp, however the levels were too low to be detected. We suspect this is because infection by HBV is relatively inefficient in this system. However, MOV10 over-expression significantly reduced HBV pgRNA (Figure 5B) and DNA levels (Figure 5C) compared to the parent HepG2-NTCP-3E8 infected cells. Collectively, these results suggest that the over-expression of MOV10 diminishes the HBV infection of HepG2-NTCP-3E8 cells.

### 3.5. HBV Pre-Genomic RNA Co-Immunoprecipitated with MOV10

**Figure 6:**
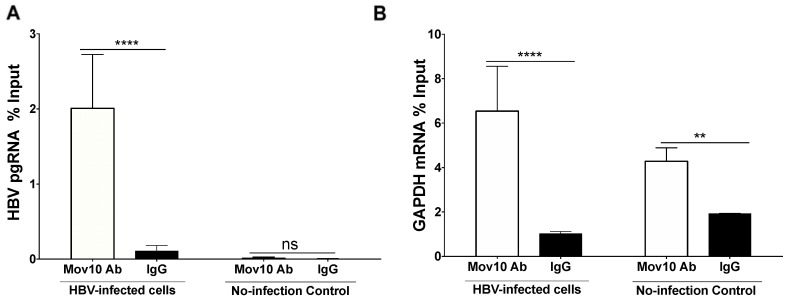
HBV pre-genomic RNA co-immunoprecipitated with MOV10. (**A**) HepG2-NTCP clone 7 cells were infected with HBV and harvested after five days. Immunoprecipitation was performed with an antibody against human MOV10 in the HBV-infected cells and in the non-infected control cells. SYBR Green-based RT-qPCR was done using primers against HBV pre-genomic RNA. (**B**) Human GAPDH mRNA co-immunoprecipitated with MOV10. SYBR Green-based RT-qPCR was done using primers against human GAPDH mRNA. (*n* = two independent experiments, ** *p* = 0.0015, **** *p* < 0.0001, two-way ANOVA Sidak’s multiple comparisons test).

As MOV10 is an RNA helicase, we wished to determine whether MOV10 interacts with HBV pgRNA. HBV pgRNA (2% of input samples) was successfully pulled-down with MOV10 (*p* < 0.0001, two-way ANOVA Sidak’s multiple comparisons test) in HBV-infected HepG2-NCTP clone 7 cells at five days post-infection. As expected, HBV pgRNA was at background level (<0.2% of input) in the non-infected cells as well as when non-specific mouse IgG was used for the pull-down (Figure 6A). These data indicate that MOV10 interacts with HBV pgRNA.

To address whether MOV10 interacts with HBV pgRNA specifically, or cellular RNAs in general, the RT-qPCR was repeated using the same samples and primers against human GAPDH mRNA (Figure 6B). GAPDH mRNA was successfully pulled-down with MOV10 from both HBV-infected samples and the no-infection controls. MOV10 IP samples yielded 6.5% and 4.2% of input in the HBV-infected and non-infected samples, respectively. The high signals observed with GAPDH mRNA in both anti-MOV10 and non-specific IgG samples likely reflect the high abundance of this RNA in the cell. The signal-to-noise ratio (MOV10 IP/IgG IP) was 6-fold for GAPDH mRNA and 20-fold for HBV pgRNA in HBV-infected samples.

### 3.6. No Specific Interaction Between MOV10 and HBV Core Protein

**Figure 7:**
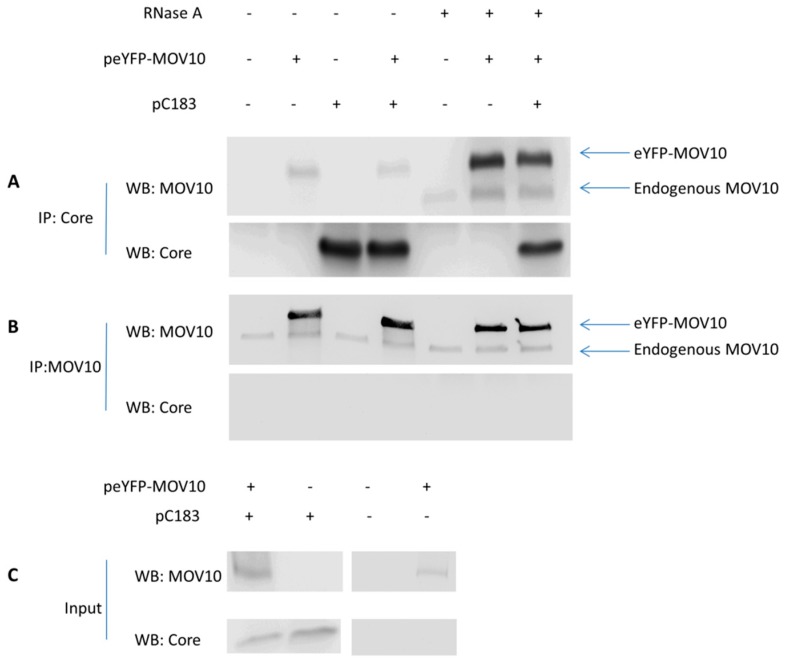
No specific interaction between MOV10 and HBV core protein. 293T cells were transfected with pC183 and peYFP-MOV10, pC183 and peYFP-empty, peYFP-MOV10 and peYFP-empty, or peYFP-empty only. Where indicated, lysates were pre-treated with RNase A prior to immunoprecipitation with the indicated antibodies. Immunoprecipitates were analyzed by western blotting using the indicated antibodies. (**A**) Proteins in lysates were immunoprecipitated with the anti-HBV core antibody. Immunoprecipitates were analyzed by western blotting using the anti-MOV10 and anti-HBV core antibodies. (**B**) Proteins in lysates were immunoprecipitated with the anti-MOV10 antibody. Immunoprecipitates were analyzed by western blotting using anti-MOV10 and anti-HBV core antibodies. (**C**) Western blotting showing detectable levels of MOV10 and HBV core proteins in tested lysates.

We also investigated whether MOV10 interacts with HBV core protein by co-immunoprecipitation (Figure 7). For this purpose, we used plasmids: (pC183) for HBV core protein expression, eYFP-MOV10 for eYFP-tagged MOV10 expression, and eYFP-empty plasmid. We transfected 293T cells with pC183 and eYFP-MOV10, pC183 and eYFP-empty, eYFP-MOV10 and eYFP-empty, or eYFP-empty only. YFP-tagged MOV10 was used to distinguish the endogenous and exogenous MOV10. After immunoprecipitation with the anti-HBV core antibody (Figure 7A), we analyzed the immunoprecipitates using the anti-MOV10 antibody for immunoblotting. Our results show the appearance of exogenous MOV10 bands (eYFP-tagged, upper bands) in all eYFP-MOV10 transfected samples, even in samples that were not transfected with pC183, which demonstrates non-specific pull-down of MOV10. We also noticed that samples treated with RNase A prior to immunoprecipitation showed MOV10 bands with increased intensity. Faint endogenous MOV10 bands (lower bands) were observed in all RNase A treated samples, even those that were not transfected with pC183. To confirm our results, we also used the anti-MOV10 antibody for immunoprecipitation (Figure 7B), followed by analyzing the immunoprecipitates using the anti-HBV core antibody for immunoblotting. We were able to immunoprecipitate both endogenous and exogenous MOV10, but could not detect co-immunoprecipitation of the HBV core protein, suggesting that the HBV core protein does not interact directly with eYFP-MOV10. Lysates were also analyzed by western blotting to demonstrate the presence of MOV10 and HBV core proteins at detectable levels in tested samples (Figure 7C). Collectively, these data indicate that there is no specific interaction between MOV10 and the HBV core protein.

### 3.7. MOV10 Over-Expression Does Not Induce Degradation of HBV pgRNA

**Figure 8:**
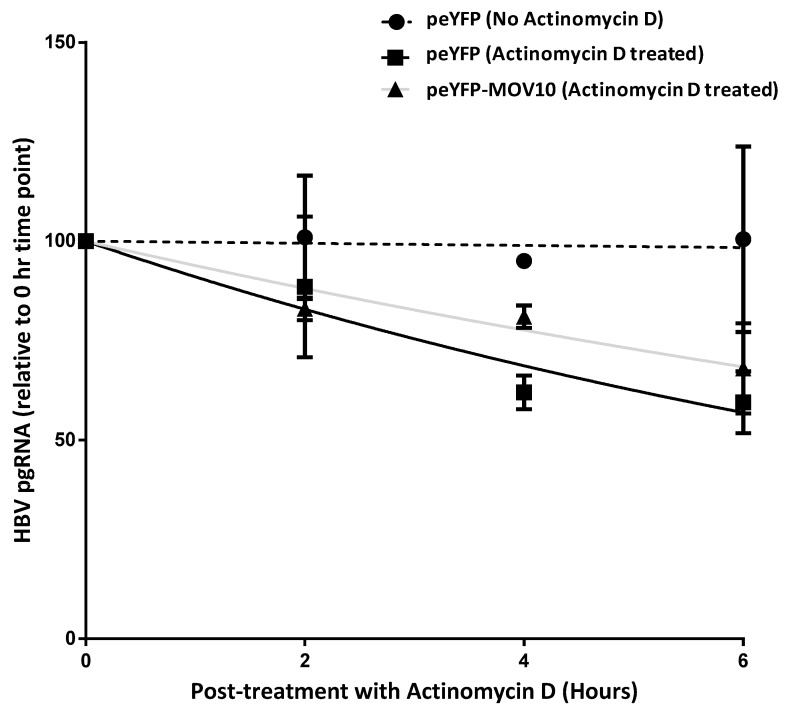
MOV10 over-expression does not induce the degradation of HBV pgRNA. 293T cells were transfected with HBV core-deficient plasmid (Cp-deficient pHBV) and peYFP-MOV10 or peYFP-empty (eYFP) plasmids. After 48 h, the cells were treated with 5 µg/mL Actinomycin D. The cells were harvested at 0, 2, 4, 6 h after Actinomycin D treatment. Total RNA was extracted and HBV pgRNA levels were determined relative to the 0 h time point. RPL18A mRNA was used as a normalization control. Single phase decay curves are shown. The dotted line represents cells transfected with Cp-deficient pHBV and peYFP in the absence of Actinomycin D. The solid black line represents cells transfected with Cp-deficient pHBV and peYFP in the presence of Actinomycin D. The grey line represents cells transfected with Cp-deficient pHBV and peYFP-MOV10 in the presence of Actinomycin D. Data represent mean ± standard deviation from two independent experiments.

As described above, we were able to demonstrate an interaction between HBV pgRNA and MOV10. To investigate whether MOV10 exerts its restriction effect on HBV replication through an induction of HBV pgRNA degradation, we measured pgRNA stability in the absence of Cp and the presence or absence of MOV10. These experiments were performed in the absence of Cp to avoid encapsidation of pgRNA, which might otherwise confound the results. 293T cells were transfected with HBV core-deficient plasmid (Cp-deficient pHBV) and eYFP-MOV10 or Cp-deficient pHBV and peYFP-empty (eYFP) plasmids in the presence or absence of Actinomycin D. Actinomycin D is a transcription inhibitor, used here to prevent the synthesis of pgRNA and allow the determination of the decay rate. The level of HBV pgRNA was determined at intervals from 0 to 6 h after Actinomycin D treatment. RPL18A mRNA was used as a normalization control, as RPL18A mRNA has a half-life of ~20 h in HEK293 cells, and is not targeted by MOV10 [20]. The levels of HBV pgRNA in the presence and absence of Actinomycin D were determined to confirm the efficiency of Actinomycin D in inhibiting transcription; a gradual decrease in HBV pgRNA level was apparent after Actinomycin D treatment (Figure 8, the dotted black line versus the solid black line). The effect of MOV10 over-expression on HBV pgRNA degradation rate was determined in the presence of Actinomycin D (Figure 8, the grey line versus the black line). Our results suggest that MOV10 over-expression did not increase HBV pgRNA degradation rate. The decay rate appeared slightly faster in the absence of MOV10 (rate constants with eYFP and eYFP-MOV10 of 0.094 and 0.063 h^−1^, respectively), however, the difference did not achieve statistical significance.

## 4. Discussion

MOV10 protein, a DExD-box RNA helicase, has been shown to be a component of P-bodies [19]. MOV10 is also involved in RISC activity [21]. Thus, MOV10 has an important role in RNA processing. Previous studies showed the importance of MOV10 as an antiretroviral host factor. Although HBV is similar to retroviruses in that it replicates through an RNA intermediate via a reverse transcription process, the role that MOV10 plays in HBV replication has not yet been extensively studied. A single manuscript reported that changes in MOV10 affect HBV DNA in HepG2 cells (and the HepG2.2.15 derivative cell line) [18]. However, the role that MOV10 has on HBV replication in an infection system is unknown. In addition, the mechanism by which MOV10 affects HBV is unknown.

Here, we studied the relationship between MOV10 expression levels and HBV gene expression. We used a variety of systems, including stably transfected cells, transient transfection, and, importantly, infection. In all systems, the suppression of MOV10 led to elevated levels of the HBV core protein, pgRNA, and DNA. These findings suggest that MOV10 inhibits HBV gene expression. Consistent with this hypothesis, MOV10 over-expression in the infectable HepG2-NTCP-3E8 cell line decreased HBV pgRNA and intracellular HBV DNA levels following infection, again suggesting that MOV10 inhibits HBV. A previous report tested the effect of MOV10 on HBV transient and stable expression systems [18]. Like us, they reported that high-level over-expression of MOV10 could inhibit HBV expression. However, they found that low-level MOV10 over-expression enhanced HBV expression and that MOV10 down-regulation decreased HBV mRNA and HBV antigens. These results are not consistent with our findings. While we find that MOV10 is an antagonist of HBV, Ma et al. reported the variable effects of different amounts of MOV10 on HBV [18]. While the reasons for this discrepancy are not fully understood, it is possible that the outcome of MOV10 silencing or over-expression depends on the cells used, and in particular on their endogenous levels of MOV10, which may be affected by unknown co-factors. In our study, we tested the levels of intracellular HBV pgRNA, DNA, and core protein, while Ma et al. tested secreted HBV antigens and HBV DNA in the supernatant and intracellular HBV mRNA [18]. These methodological differences may contribute to the differences between our findings and those of Ma et al. [18], however, both groups measured intracellular HBV RNA, with different outcomes. Similar unresolved discrepancies were found by the different research groups who studied the role of MOV10 in retroviral replication and infectivity. These differences may be due to variable levels of MOV10 expression or due to a possible complicated role of MOV10 during the different stages in the virus life cycle [22,23,24].

In our attempts to understand the mechanism by which MOV10 exerts its restriction activity against HBV replication, we provided evidence that MOV10 affects pgRNA levels, and consequently protein and DNA levels, but there was no induction of pgRNA degradation by MOV10 over-expression. This is reminiscent of reports that MOV10 reduces HIV-1 Gag expression, but does not reduce gag RNA steady-state level or gag RNA turnover rate [45].

It has been reported that MOV10 exerts some of its activity against HIV-1 following incorporation into virions [23,46]. We observed no direct interaction between MOV10 and the HBV core protein using co-immunoprecipitation technique in 293T cells, however, we cannot eliminate a low affinity interaction, or an interaction mediated through a second, hepatocyte-specific factor. The lack of direct interaction between MOV10 and the HBV core protein does not exclude the possibility that MOV10 can be packaged into HBV cores, possibly through the interaction of MOV10 with HBV pgRNA. Incorporation into HBV particles and restriction of HBV has been demonstrated for the APOBECs, which, like MOV10, are RNA binding proteins that are localized to p-bodies [25,47,48].

## 5. Conclusions

We have demonstrated in multiple cell lines and in an HBV infection system that MOV10 down-regulation enhances HBV gene expression, while MOV10 over-expression suppresses HBV. We also showed that MOV10 interacts with HBV pgRNA. These findings support an inhibitory activity for MOV10, suppressing HBV infection and replication, mediated against the HBV pgRNA, and possibly other HBV RNAs.

## Abbreviations

APOBEC3G	Apolipoprotein-B-mRNA-editing enzyme catalytic polypeptide-like 3G
cDNA	Complementary DNA
cccDNA	Covalently closed circular DNA
CT	Cycle threshold
DMSO	Dimethyl sulfoxide
DMEM	Dulbecco’s Modified Eagle’s Medium
DPBS	Dulbecco’s Phosphate-Buffered Saline
FBS	Fetal bovine serum
G418	Geneticin
GAPDH	Glyceraldehyde 3-phosphate dehydrogenase
pC183	HBV core expressing plasmid
HBV	Hepatitis B virus
HCV	Hepatitis C virus
HDV	Hepatitis D virus
h	Hour
hpt	Hours post-transfection
HIV-1	Human immunodeficiency virus type 1
IP	Immunoprecipitation
MOV10	Moloney leukemia virus 10
siMOV10	MOV10 siRNA
MLV	Murine leukemia virus
NEAA	Non-essential amino acids
sicontrol	Non-targeting control siRNA
pHBV	pCMV-HBV-LE-II
pgRNA	Pre-genomic RNA
PEG	Polyethylene glycol
PBS	Phosphate-Buffered Saline
rcDNA	Relaxed-circular DNA
RT	Room temperature
SIV	Simian immunodeficiency virus
SDS-PAGE	Sodium dodecyl sulfate polyacrylamide gel electrophoresis
TBST	Tris Buffered Saline with 0.1% Tween 20
